# Pretransitional Effects of the Isotropic Liquid–Plastic Crystal Transition

**DOI:** 10.3390/molecules26020429

**Published:** 2021-01-15

**Authors:** Aleksandra Drozd-Rzoska, Szymon Starzonek, Sylwester J. Rzoska, Joanna Łoś, Zdravko Kutnjak, Samo Kralj

**Affiliations:** 1 Institute of High Pressure Physics Polish Academy of Sciences, ul. Sokołowska 29/37, 01-142 Warsaw, Poland; arzoska@unipress.waw.pl (A.D.-R.); sylwester.rzoska@unipress.waw.pl (S.J.R.); joalos@unipress.waw.pl (J.Ł.); 2 Condensed Matter Physics Department, Jožef Stefan Institute, Jamova 39, 1000 Ljubljana, Slovenia; zdravko.kutnjak@ijs.si (Z.K.); samo.kralj@um.si (S.K.); 3 Faculty of Natural Sciences and Mathematics, University of Maribor, Koroška 160, 2000 Maribor, Slovenia

**Keywords:** plastic crystal, melting, pretransitional behavior, nonlinear dielectric effect, dielectric constant, Mossotti Catastrophe

## Abstract

We report on strong pretransitional effects across the isotropic liquid–plastic crystal melting temperature in linear and nonlinear dielectric response. Studies were carried out for cyclooctanol (C_8_H_16_O) in the unprecedented range of temperatures 120 K < *T* < 345 K. Such pretransitional effects have not yet been reported in any plastic crystals. Results include the discovery of the experimental manifestation of the Mossotti Catastrophe behavior, so far considered only as a hypothetical paradox. The model interpretations of experimental findings are proposed. We compare the observed pretransitional behavior with the one observed in octyloxycyanobiphenyl (8OCB), typical liquid crystal (LC), displaying a reversed sequence of phase transitions in orientational and translational degrees of order on varying temperature. Furthermore, in its nematic phase, we demonstrate first-ever observed temperature-driven crossover between regions dominated by isotropic liquid and smectic A pretransitional fluctuations. We propose a pioneering minimal model describing plastic crystal phase behavior where we mimic derivation of classical Landau-de Gennes-Ginzburg modeling of Isotropic-Nematic-Smectic A LC phase behavior.

## 1. Introduction

Orientationally disordered crystals (ODICs) [1,2,3], also referred to as plastic crystals (PC), and liquid crystals (LCs) [4,5] are mesophases that can exist between the isotropic liquid and solid crystalline phases. Their unique properties are associated with the dominance of a single element of symmetry. For example, the nematic LC phase, representing the simplest LC configuration, is characterized by the orientational order and the translational disorder [4,5]. On the contrary, ODICs exhibit translational order and orientational disorder [1,2,3]. These configurations can be considered as convenient, simple, and test-bed systems, from which fundamentals of the impact of different sequences of orientational and translational phase ordering on material properties could be extracted [1,2,3,4,5,6,7,8,9]. In particular, isotropic liquid (I)-mesophase (M) transitions are associated with melting/freezing of only a single element of symmetry.

However, existing literature suggests significantly different temperature-driven pretransitional behavior of I-ODIC and I-LC phase transitions. In the latter case, cooling towards the LC mesophase is associated with strong pretransitional effects in a wide temperature window within the liquid phase. Indeed, strong pretransitional changes of the Kerr effect (KE) [4,5,6,10,11] and Cotton Mouton effect (CME) [4,5,6,12,13] in the isotropic liquid phase of nematic LCs inspired development of the Landau-de Gennes (LdG) model [4,5,6,14,15], representing important cornerstone for theoretical description of LCs [3,4,5,6,7] and soft matter physics in general [16,17]. On the contrary, in ODIC-forming systems the existing evidence indicates negligible weak pretransitional effect [18,19,20,21,22,23,24,25,26,27,28,29,30,31,32,33,34,35], suggesting the behavior found in systems exhibiting classical liquid-crystal discontinuous phase transitions [36,37,38,39,40,41]. The only exception is reported for the optical Kerr effect (OKE) pretransitional effect of in *p*-terphenyl (1993, [42]), which could be rather linked to the hidden isotropic-nematic (I-N) phase transition.

This report shows the first-ever experimental evidence of strong pretransitional effects for the isotropic liquid-ODIC (I-PC) phase transitions, extending over a wide temperature window. Such behavior is observed in dielectric constant and its strong electric field counterpart, the nonlinear dielectric effect (NDE), both surprisingly not tested so far. Phase behavior was probed in the extreme range of temperatures in cyclooctanol (C_8_OH), one of the most classical ODIC-forming materials [18,19,20,21,22,23,24,25,26,27,28,29,30,31,32,33,34,35]. As a reference LC system, we studied a rod-like LC material, *n*-octyloxycyanobiphenyl (8OCB), which also revealed new aspects of pretransitional effects. Emerging similarities can offer a path for a common description of the melting phenomenon in LC- and ODIC-forming materials.

The paper is organized as follows. In Section 2, we present experimental results in samples displaying liquid crystalline and plastic crystal phase ordering. Results are discussed in Section 3. In Section 4, the experimental methods used in our studies are introduced. In the last section, we summarize results. In Appendix A main, the derivation of the classical Claussius-Mossotti equation is summarized. Minimal models comparing liquid crystalline and plastic crystal phase behavior and derivation are given in Supplementary Materials.

## 2. Results

We measured linear and nonlinear dielectric responses in representative LC and ODIC materials, focusing on pretransitional phenomena. We first present the characteristic dielectric response of liquid crystalline 8OCB. Then, a detailed study of plastic crystalline C_8_H_16_O is subsequently presented. 

### 2.1. LC-Forming Materials: The Case of 8OCB

When passing the isotropic liquid (I)–nematic (N) clearing (melting) temperature solely the orientational ordering freezes/melts, whereas the translational fluid-like disarrangement remains. For phase transitions associated with smectic mesophases also some elements of a limited translational ordering appear [5,6]. Dielectric constant measurements reveal efficient macroscopic consequences of melting/freezing in LC materials. This physical property fingerprints effective dipole-dipole arrangements. Note that rod-like LC mesophases exhibit the head-to-tail invariance of the nematic director field n, which determines local uniaxial orientational LC order. If such LC compound molecule contains the permanent dipole moment parallel to the long molecular axis, then ±n invariance in the nematic phase favors cancellation of dipole moments [5,6]. The broadband dielectric spectroscopy is the basic tool also for determining dielectric constant as the stationary, frequency-independent, domain of the real part of dielectric permittivity (ε’(f)). The imaginary part of dielectric permittivity enables tests of dynamics associated with permanent dipole moments via the structural (primary, alpha) relaxation time determined from related loss curves peaks $\tau =1/2\pi f_{peak}$ [43]. 

**Figure 1:**
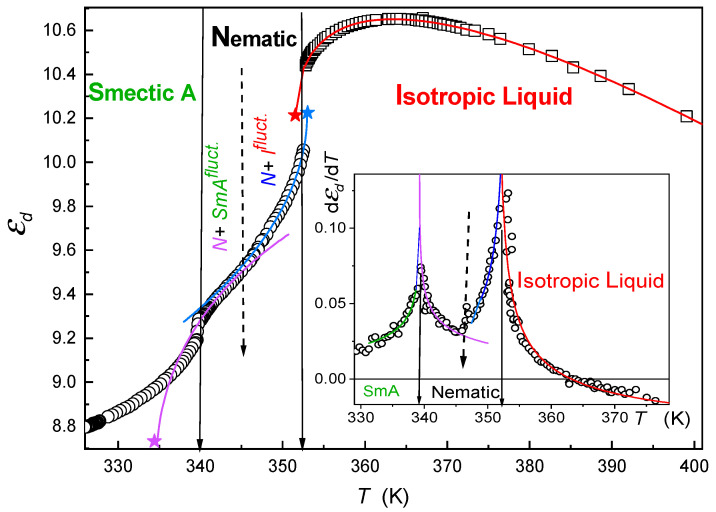
The temperature evolution of dielectric constant in the isotropic liquid and liquid crystalline mesophases of 8OCB. The latter is presented by the mean dielectric constant $\varepsilon_{d}=\left(2\varepsilon_{⊥}+\varepsilon_{∥}\right)/3$. Solid curves represent best fits using Equations (2)–(4). Stars denote the hypothetical extrapolated temperatures of continuous phase transitions. Solid arrows indicate temperatures of discontinuous I-N and N-SmA phase transitions. The dashed arrow indicates the crossover temperature in the nematic phase between domains dominated by isotropic liquid and SmA pretransitional fluctuations. The inset shows the distortion’s sensitive behavior of the derivative of mean dielectric constant, supporting the picture emerging from the main plot.

Figure 1 shows the temperature evolution of dielectric constant in the broad range of temperatures for 8OCB, encompassing isotropic, nematic and SmA phase regimes. Temperature changes of dielectric constant in the isotropic liquid phase are well portrayed by the relation [44]

(1) $$\varepsilon \left(T\right)=\varepsilon^{*}+a_{\varepsilon }\left(T-T_{I}^{*}\right)+A_{\varepsilon }{\left(T-T_{I}^{*}\right)}^{\left(1-\alpha \right)}$$

describing dielectric response on lowering temperature. It holds that $T>T_{I-N}=T_{I}^{*}+\Delta T_{I}^{*}$, where $T_{I}^{*}$ is the temperature of the hypothetical continuous phase transition, $T_{I-N}$ determines the isotropic-nematic transition, and $\Delta T_{I}^{*}$ is the metric of the discontinuity of the phase transition; $\varepsilon^{*},$ $a_{\varepsilon },$ and $A_{\varepsilon }$ are phenomenological constants and α≈0.5 is the critical exponent. Note that the temperature window described well with Equation (1) extends up to at least $T_{I-N}+70K$.

In the nematic phase, rod-like dipolar LC molecules are easily oriented with an external field; then, components of dielectric constant for molecules predominantly oriented perpendicularly ($\varepsilon_{⊥}$) and parellely ($\varepsilon_{∥}$) in the respect to the measuring electric field should be considered [5,6]. This was made by the strong magnetic field (*B* ~1 T). The dielectric anisotropy $\Delta \varepsilon =\varepsilon_{\left|\right|}-\varepsilon_{⊥}$ is commonly used to measure the order parameter in the nematic phase. In the nematic phase it holds that $\Delta \varepsilon \left(T\right)=\varepsilon^{*}+B{\left(T_{N}^{*}-T\right)}^{\beta }$: in 8OCB, the classical tricritical value of the exponent β≈1/4 was reported [45]. Furthermore, the diameter of dielectric constant $\varepsilon_{d}=\left(2\varepsilon_{⊥}+\varepsilon_{∥}\right)/3$ is often of interest because it is related to the mean dielectric response of a dielectric constant in the nematic phase [5,6]. Its evolution in the nematic phase can be well portrayed by the relations [46]

(2) $$\varepsilon_{d}\left(T\right)=\varepsilon_{N}^{*}+a_{N}\left(T_{N}^{*}-T\right)+A_{N}{\left(T_{N}^{*}-T\right)}^{\left(1-\alpha \right)},$$

(3) $$\varepsilon_{d}\left(T\right)=\varepsilon_{SmA}^{*}+a_{SmA}\left(T-T_{SmA}^{*}\right)+A_{SmA}{\left(T-T_{SmA}^{*}\right)}^{\left(1-\alpha \right)}.$$

Here, Equations (2) and (3) are valid for temperature variations driving by N→I and N→SmA phase transitions, taking place at $T_{I-N}$ and $T_{N-SmA}$, temperatures. Quantities $\varepsilon_{N}^{*},\;\varepsilon_{SmA}^{*},\;a_{N},A_{N},a_{SmA},A_{SmA}$ are phenomenological constants, and $T_{N}^{*}=T_{I-N}+∆T_{N}$ and $T_{SmA}^{*}=T_{N-SmA}-∆T_{SmA}$ are temperatures of hypothetical continuous phase transitions determined from extrapolations from the nematic phase. Note that Equations (1)–(3) are linked to the same value of the critical exponent α≈1/2.

**Figure 2:**
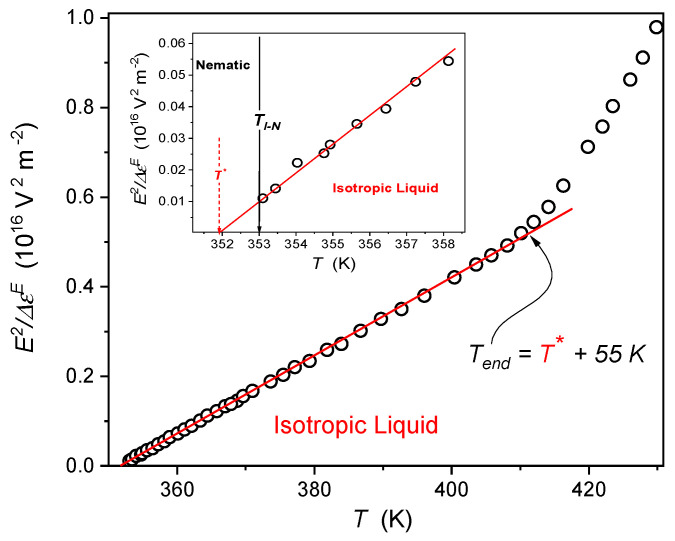
The temperature dependence of the reciprocal of nonlinear dielectric effect (NDE), strong electric field related changes of dielectric constant, in the isotropic liquid phase of liquid crystalline 8OCB. The line shows the validity of portrayal via Equation (4), extending from the Isotropic-Nematic melting temperature to *T_end_*. The inset shows the behavior in the immediate vicinity of the I-N melting (clearing) temperature, indicated by the solid arrow. The dashed arrow indicates the hypothetical continuous phase transition.

We stress that Figure 1 reveals that the nematic phase is not homogeneous, a fact which has been previously described in [46,47,48,49]. There are two domains linked to vicinities of N→I and SmA←N phase transitions. This is particularly visible for the distortions sensitive plot shown in the inset in Figure 2, which also evidences the validity of ansatz used in fitting experimental measurements via Equations (2) and (3). Namely, it holds that $\frac{d\varepsilon_{d}}{dT}\propto {\left|T-T^{*}\right|}^{-\alpha }\approx {\left|T-T^{*}\right|}^{-1/2}$, and $T^{*}$ stands either for $T_{I}^{*}$, $T_{N}^{*}$, or $T_{SmA}^{*}$, respectively. When explaining origins of pretransitional anomalies shown in Figure 1 one should indicated that in the isotropic liquid phase dielectric constant of prenematic fluctuations is much smaller than for the isotropic liquid surrounding $\varepsilon_{fluct.}<<\varepsilon_{surr.}$. Core increase of the volume occupied by prenematic fluctuation occurring for $T\to T_{I-N}$ leads to the crossover dε(T)/dT<0→dε(T)/dT>0. For the N→I transition, the isotropic fluctuations are surrounded by the nematic background, thus $\varepsilon_{fluct.}>>\varepsilon_{surr.}$, leading to Equation (2). For SmA←N the distortion of the orientational ordering by 1-D smectic ordering causes that $\varepsilon_{fluct.}>\varepsilon_{surr.}$, yielding Equation (3).

**Figure 3:**
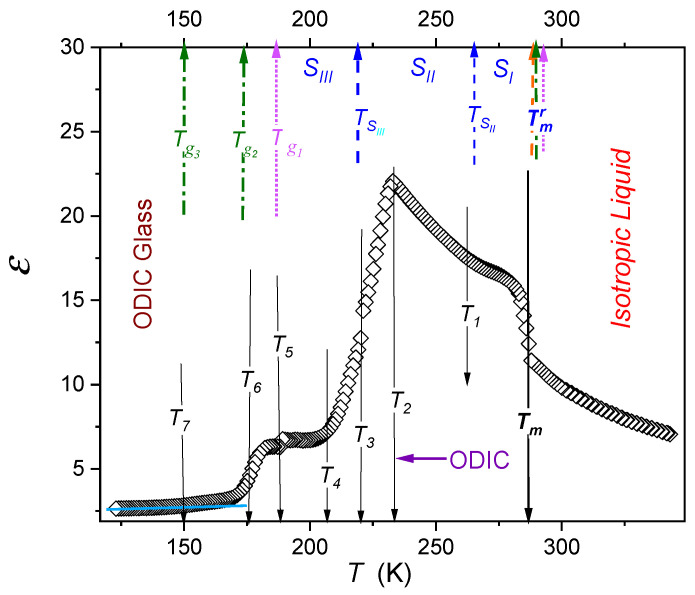
Temperature evolution of the dielectric constant in ODIC-forming cyclooctanol. Solid arrows pointing downwards show characteristic temperatures extracted from our measurements: $T_{m}=286.4$ K, $T_{1}=233$ K,$\;T_{2}=207.1$ K, $T_{3}=178.2$ K. Arrows pointing upwards are related to previously published values of relevant critical temperatures: (i) blue, dashed: from the work in [7], (ii) dotted, pink: from the work in [7], and (iii) dot-dashed, green: from the work in [8].

The temperature evolution of the strong electric field (*E*) related (nonlinear) counterpart of dielectric constant is shown in Figure 3. This magnitude, known as the nonlinear dielectric effect (NDE), exhibits an extreme pretransitional anomaly in the isotropic liquid phase of LC materials, portrayed by the following relation [48,49,50,51,52,53,54,55,56],

(4) $$\frac{\Delta \varepsilon^{E}}{E^{2}}=\frac{C}{T-T_{I}^{*}}$$

where *C* is the material constant and $T_{I}^{*}$ is the temperature of the hypothetical continuous phase transition as discussed above. 

Parallel relations describe the behavior of the intensity of the scattered light, Cotton-Mouton effect, or the Kerr effect in the isotropic liquid phase of nematic LC materials [5,6,7,8,9,10,11,12,13,14,15,16]. In fact, those studies were the inspiration for the Landau-de Gennes model (LdG), the key theoretical concepts for the physics of liquid crystals [5,6], and for the general soft matter physics [16,17]. Note that NDE is the only method for which Equation (4) also obeys for I-N, I-SmA, I-SmE, and I-N* transitions. This is associated with the fact that for NDE the measurement time scale, related to the radiofrequency of the weak measuring field $\tau_{method}>>\tau_{fl.}$ [50,51,52,53,54]. For KE, CME, or IL $\tau_{method}<<\tau_{fl.}$, what is associated with the light-related measurement frequency. Equation (5) can be derived from LdG model-based consideration and also from the expression originally derived to model NDE and KE pre-critical effects on approaching the critical consolute point in binary mixtures of limited miscibility or the gas-liquid critical point [53,54,55]:

(5) $$\frac{\Delta \varepsilon^{E}}{E^{2}}\propto \chi \langle \Delta M^{2}\rangle {}_{V}$$

where $\langle \Delta M^{2}\rangle {}_{V}\propto {\left|T-T_{C}\right|}^{2\beta }$ stands for the averaged square of the order parameter fluctuations and $\chi =\chi_{0}{\left|T-T_{C}\right|}^{-\gamma }$ denotes the compressibility (order parameter related susceptibility). The critical temperature $T_{C}$ is associated with the critical temperature of a continuous phase transition. For the isotropic phase of LC materials, it then holds that $T_{C}=T_{I}^{*}$.

In the isotropic liquid phase, the mean-field approximation works relatively well, owing to the elongated and rod-like form of LC molecules which increases the number of neighboring molecules. It is notable that both compressing and strong electric fields can change the volume/shape of pretransitional fluctuations but cannot influence dielectric constant related to prenematic fluctuations. Consequently, in the isotropic liquid phase, it is reasonable to set $\langle \Delta M^{2}\rangle {}_{V}\propto {\left(\Delta \varepsilon \right)}^{2}=const$ and $\chi =\chi_{0}/{\left(T-T^{*}\right)}^{-\gamma }$ with the classical exponent γ=1, which leads to Equation (4). Note that in the isotropic phase, changes in dielectric constant are well described using Equation (1) up to at least 70 K above the clearing temperature. For NDE, the pretransitional effect described by Equation (4) persists until $T_{nde}\approx T_{I}^{*}+40\;K$ [49,50,51,52,53]. This can be linked to the reduction of pre-mesomorphic fluctuations to 2–3 molecules, making their detection by methods directly coupled to their presence, such as NDE, impossible.

### 2.2. ODIC-Forming Materials: The Case of Cyclooctanol

For plastic crystalline materials when cooling below the I-ODIC freezing/melting temperature $T_{m}$ the translational ordering appears but the orientational freedom remains. Experimental evidence from previously published results [18,19,20,21,22,23,24,25,26,27,28,29,30,31,32,33] seems to be clear: there are no pretransitional behavior in the surrounding of $T_{m}$, contrary to the discussed above case of LC materials. Note that on cooling ODIC forming materials most often terminate in the orientationally disordered solid glass state. Consequently, studies of such systems are focused mainly on the glass transition problem, for which the enormous shift of the primary relaxation time from pico/nanoseconds to $\tau \left(T_{g}\right)\approx 100$ s at the glass temperature $T_{g}$ is the key artifact. Consequently, the broad band dielectric spectroscopy (BDS) is an appropriate experimental research tool to study main features of ODICs.

**Figure 4:**
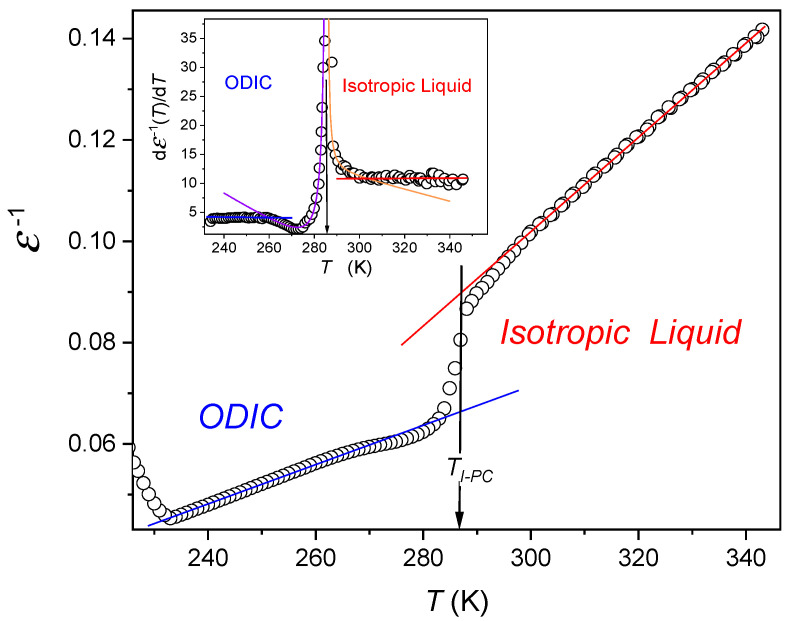
The evolution of the reciprocal of dielectric constant in cyclooctanol. The plot is based on experimental shown in Figure 4. In the isotropic liquid and in the notable part of the ODIC phase, the $\varepsilon^{-1}$ temperature dependence exhibits linear temperature dependence, suggesting the validity of Equation (6a) with extrapolated singular temperatures $T^{+}=195$ K (blue line) and $T^{+}=115$ K (red line). The derivative-based and distortions sensitive analysis in the upper inset confirms the mentioned behavior, also revealing a distortion in the very immediate vicinity of $T_{m}$. It can be portrayed by Equation (6b), what is shown by solid curves.

Figure 4 shows the temperature evolution of dielectric constant of cyclooctanol (C_8_OH), one of the most classical ODIC-forming materials, in the broadest ever studied temperature range covering also the liquid phase from ~120 K up to $T_{m}+70$ K. Such an extreme range has to be associated with the qualitative shift of the structural (primary) relaxation time from τ≈5 ps to $\tau \left(T_{g}\right)\approx 100$ s, which also leads to the shift of the location of the static domain of $\varepsilon^{\prime }\left(f\right)=\varepsilon$ from ~1 MHz to even ~1 Hz domain. This shift was considered for the experimental data discussed below. Figure 4 shows the temperature evolution of dielectric constant for cyclooctanol. Previous investigations focused mainly on heat capacity and BDS based dielectric relaxation studies and led to the identification of three possible ODIC phases (*S_I_*, *S_II_*, *S_III_*) and suggested three possible glass temperatures (*T_g1_*, *T_g2_*, *T_g3_*). These values are shown in Figure 4 by dashed arrows, oriented upwards. The scan of the dielectric constant sheds new light on ODIC mesomorphism and characteristic temperatures in C_8_OH. There are also characteristic temperatures not specified so far, such as $T_{2}$ associated with the sharp crossover dε/dT>0←dε/dT<0 and $T_{4}$ linked to the change dε/dT≈0←dε/dT>0. The question arises for $T_{S_{II}}$, indicated earlier as the phase transition between *S_I_* and *S_II_* ODIC phase. As shown below, $T_{S_{II}}\approx T_{1}$ can be associated with crossover between two pretransitional domains in the ODIC phase. Remember a similar crossover occurring in the nematic phase of liquid crystalline 8OCB, see Figure 1. Changes of ε(T) values can be associated with the increase/decrease of the freedom of permanent dipole moment for following changes in the electric field. The behavior described by dε/dT<0 or dε/dT>0 indicates the preference for parallel or antiparallel arrangements of permanent dipole moments [42]. 

Figure 4 shows the temperature evolution of the reciprocal of dielectric constant, which reveals simple forms of the temperature evolutions in the isotropic liquid for $T>T_{m}$ and in the ODIC phase for $T_{2}<T<T_{m}$:

(6a) $$\varepsilon^{-1}\left(T\right)=b+aT\to \varepsilon \left(T\right)\propto \frac{A}{T-T^{+}},$$

where *a*, *b*, and *A* are constants and $T^{+}$ denotes the extrapolated singular temperature; the linear regression analysis yields in the liquid phase $T^{+}=195$ K (A=1078 K), and $T^{+}=115$ K (A=2490 K) in the ODIC phase.

Such a description is also validated by the distortions-the sensitive derivative plot $d\varepsilon^{-1}\left(T\right)/dT$ shown in the upper inset in Figure 4. Its parameterization shows that in the immediate vicinity of $T_{m}$ the additional critical-like behavior appears:

(6b) $$\frac{d\varepsilon }{dT}\propto {\left(T-T^{*}\right)}^{-1.5}$$

for $T=T_{m}\pm 10K$ and singular temperatures T* = 276.0 K for I→ODIC and T* = 293.8 for ODIC→I transitions.

**Figure 5:**
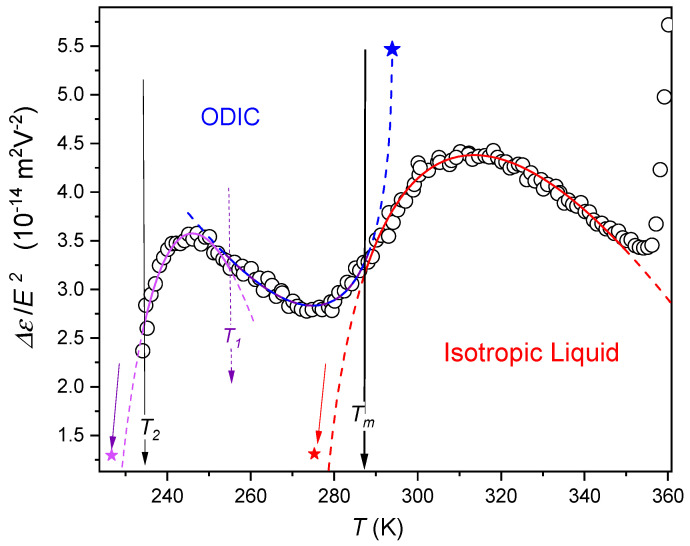
The temperature dependence of nonlinear dielectric effect (NDE) in the liquid and ODIC phases of cyclooctanol. Solid curves are related to Equation (7) with *T_m_* = 287.5 K (T* = 276 K), *T*_1_ = 254.5 K (T* = 293.8), *T*_2_ = 234 K (T* = 220 K). Fitting parameters are given in Table 1.

The nonlinear dielectric effect (NDE) describes changes of dielectric constant under the strong electric field. It can directly detect the appearance of collective phenomena, such as pretransitional fluctuations. This sensitivity is associated with different interactions of the collective species (fluctuation) and the surrounding background with the strong electric field. Figure 5 presents results of the first-ever NDE measurements in the liquid and ODIC phases of aplastic crystal-forming material, C_8_OH in the given case.

There is a strong and wide temperature range pretransitional effect in the liquid phase, extended even up to approximately $T_{x}\approx T_{m}+50$ K, well portrayed by the relation

(7) $$\frac{\Delta \varepsilon }{E^{2}}\left(T\right)=\frac{\Delta \varepsilon }{E^{2}}\left(T^{*}\right)+A_{\Delta }\left|T-T^{*}\right|+B_{\Delta }{\left|T-T^{*}\right|}^{\varphi }$$

where the exponent φ=1/2 and $T^{*}$ is the singular temperature, possibly that associated with a hypothetical hidden continuous phase transition. The value of $\Delta T^{*}\approx 12K$ can be considered as the metric of ODIC⥂I phase transition discontinuity. Note that this value is similar to ones detected for I-SmE transitions in liquid crystalline material via NDE measurements [45,53]. It is worth recalling that the SmE phase the orientational arrangement is assisted by so complex translational structure that such materials are encountered both to liquid crystalline and generalized plastic crystalline materials [6,53]. 

Note that for ODIC←I transition approximately the same value of the singular temperature $T^{*}\approx 276$ K were obtained from NDE studies (Equation (7)) and from subtle changes of dielectric constant in the immediate vicinity of $T_{m}$ (Equation (6b)). Equation (7) fitting parameters are presented in Table 1. The comparison of discontinuities of phase transitions yields $\Delta T^{*}\left(ODIC\to I\right)\approx \left(1/2\right)\Delta T^{*}\left(I\to ODIC\right)$. This value is similar to the ones noted for the I→N and N→I phase transitions in liquid crystalline materials [48].

## 3. Discussion

Figure 4 and Figure 5 reveal strong pretransitional anomalies in the liquid and plastic crystal phases of cyclooctanol. We claim it can announce a phase transition in orientational order of cyclooctanol molecules. This ordering is also linked to the collective ordering of permanent dipoles that the molecules host. Below we illustrate that this phase transition results in the Mossotti catastrophe (see Appendix A) [7,20,55]. The latter is fingerprinted in the observed critical pretransitional behavior in dielectric responses. 

We originate from the classical Claussius-Mossotti equation, which relates the macroscopic dielectric response with microscopic dipolar properties (see Appendix A). We set that the system consists of two different types of electric dipoles, to which we refer as collective and non-collective dipoles, respectively. The former (latter) behave as effectively coupled (non-coupled) ensemble. The total electric polarization P of the system is then expressed as

(8) $$P\sim n^{\left(c\right)}p^{\left(c\right)}+n^{\left(n\right)}p^{\left(n\right)}=n^{\left(c\right)}\alpha^{\left(c\right)}E_{loc}^{\left(c\right)}+n^{\left(n\right)}\alpha^{\left(n\right)}E_{loc}^{\left(n\right)},$$

where the superscripts ^(c)^ and ^(n)^ refer to collective and non-collective contributions, α is the polarizability constant, *n* labels volume density of electric dipoles, ***p*** stands for an average electric dipole experiencing the local electric field $E_{loc}.$ Furthermore, we assume $E_{loc}^{\left(c\right)}\sim E_{loc}^{\left(n\right)}\equiv E_{loc}=E_{loc}e_{E},$ where $\left|e_{E}\right|=1$.

We set that the collective dipolar response exhibits temperature driven phase transition at the critical temperature $T_{c}$. We define the orientational order parameter amplitude $m_{o}$ of the phase transition by

(9) $$m_{o}=<e.e_{E}>\;=<cos\theta >\;.$$

where e denotes a temporal orientation of a dipole, <….> stands for the ensemble average, and θ is the angle between the unit vectors e and $e_{E}$. Therefore, isotropic distribution of e or their strict alignment along the symmetry breaking direction $e_{E}$ results in $m_{o}$ = 0 or $m_{o}$ = 1, respectively. Consequently, an average collective electric dipole can be expressed as

(10) $$p^{\left(c\right)}=p_{0}^{\left(c\right)}m_{o}.$$

The corresponding free energy density *f* describing the critical behavior is given by $f=f_{c}+f_{E}$. It consists of the condensation ($f_{c}$) and the external field ($f_{E}$) contributions:

(11a) $$f_{c}\sim a_{0}\left(T-T^{*}\right)m_{0}^{2}-bm_{0}^{4}+cm_{0}^{6}$$

(11b) $$f_{E}\sim -n^{\left(c\right)}E_{loc}p_{0}^{\left(c\right)}m_{o}.$$

Here, we use the simplest possible modeling to illustrate the mechanism yielding the observed pretransitional behavior. The more detailed description is presented in Appendix A. The condensation term describes the temperature-driven critical behavior of collective dipoles for $E_{loc}=0$. The quantities $a_{0}>0$ and *b* are the Landau expansion coefficients. For *b* > 0 the phase transition is discontinuous and $T^{*}$ determines the supercooling temperature. 

A finite value of *E* (and consequently $E_{loc}$) enforces a finite macroscopic dipolar orientational ordering in the whole temperature regime. For relatively high temperatures it holds that

(12) $$f\sim a_{0}\left(T-T^{+}\right)m_{0}^{2}-n^{\left(c\right)}p_{0}^{\left(c\right)}E_{loc}m_{o}.$$

The minimization of the above relation with respect to $m_{o}$ yields

(13) $$m_{o}=\frac{n^{\left(c\right)}p_{0}^{\left(c\right)}E_{loc}}{2a_{0}\left(T-T^{+}\right)}.$$

Considering Equation (8), it follows that $\alpha^{\left(c\right)}=\frac{n^{\left(c\right)}p_{0}^{\left(c\right)2}\;}{2a_{0}\left(T-T^{+}\right)}$. With this in mind, we generalize the classical Claussius-Mossotti equation (see Equation (A4) in Appendix A) to the case consisting of collective and non-collective dipolar contributions. By considering $n\alpha \to n^{\left(c\right)}\alpha^{\left(c\right)}+n^{\left(n\right)}\alpha^{\left(n\right)}$, it follows that

(14) $$\frac{\varepsilon -1}{\varepsilon +2}=\frac{n^{\left(n\right)}\alpha^{\left(n\right)}}{3\varepsilon_{0}}+\frac{n^{\left(c\right)2}p_{0}^{\left(c\right)2}\;}{6\varepsilon_{0}a_{0}\left(T-T^{+}\right)}\;,$$

which predicts a critical-like response for temperatures $T>T^{+}.$ The equation exhibits the Claussius-Mossotti catastrophe at $T=T^{+}$.

In the above description, we considered only the phase transition in the orientational degree of order. However, in the studied ODIC system there is a sequence of phase transitions in translational degrees of freedom in the temperature regime above $T_{c}.$ In general the translational and orientational degrees are weakly coupled (Supplementary Materials), which manifests in different measured values of $T^{+}$ above and below $T_{m},$ as evidence Figure 4. Furthermore, the above description assumes ferroelectrically ordered orientationally ordered phase. However, the ordered phase could also exhibit an anti-ferroelectric order. In this case $m_{o}$ refers to ordering in an anti-ferroelectric subdomain.

Furthermore, ODIC and 8OCB LC display reversed temperature-dependent behavior of linear and nonlinear dielectric responses in the temperature regime $T>T_{c}$ ($T_{c}\equiv T_{I-N}$ for 8OCB and $T_{c}\equiv T_{m}$ for C_8_H_16_O). In 8OCB, the low field (linear) dielectric response exhibits non-monotonic ε(T) dependence, switching from $\frac{d\varepsilon }{dT}<0$ to $\frac{d\varepsilon }{dT}>0$ behavior. On the contrary, the high field NDE (nonlinear) response monotonically increases. In ODIC one observes opposite behavior. Namely, ε(T) monotonically increases, while NDE exhibits non-monotonic temperature behavior on decreasing *T.*

A possible qualitative explanation for the observed pretransitional behaviors in 8OCB is as follows. In both the linear and nonlinear regimes, the system consists of a “sea” of strongly fluctuating (but weakly ordered by E) LC molecules within which exist “islands” (clusters) exhibiting relatively strong paranematic order. The former molecules effectively behave like non-collective system members. Their orientational order is dominated by thermal fluctuations. On the contrary, paranematic clusters correspond to relatively ordered domain regions, each characterized by an average local symmetry breaking direction $n^{\left(c\right)}$. These regions contribute to the collective response, which is dominated by nematic interactions. These favor locally parallel mutual orientation (exhibiting head-to-tail invariance) of neighboring LC molecules. We henceforth label quantities referring to non-collective and collective contributions with superscripts ^(n)^ and ^(c)^, respectively. Therefore, we express the total dielectric response as

(15) $$\varepsilon =\frac{V^{\left(n\right)}}{V}\varepsilon^{\left(n\right)}+\frac{V^{\left(c\right)}}{V}\varepsilon^{\left(c\right)},$$

where $V=V^{\left(n\right)}+V^{\left(c\right)}$ determines the total volume of a sample. On decreasing *T* the relative presence of collective contribution increases. Namely, it roughly holds $V^{\left(c\right)}\sim N\xi_{n}^{3},$ where *N* determines the number of nucleated paranematic clusters, and $\xi_{n}$ is the nematic order parameter correlation length which increases on approaching $T_{c}$.

In the linear regime, the orientational probability distribution function of $n^{\left(c\right)}$ is roughly isotropic. Consequently, we claim that for temperatures $T>T_{c}$ it holds that $\varepsilon^{\left(c\right)}<\varepsilon^{\left(n\right)}$. Namely, E-driven reorientations of electric dipoles of non-collective molecules are on average not hindered by LC molecular fields. On the other hand, a relatively larger number of paranematic clusters are oriented perpendicular to the weakly imposed symmetry breaking field E. The response of such molecules is closer to $\varepsilon_{⊥}$, where $\varepsilon_{⊥}\ll \varepsilon_{∥}$, and $\varepsilon_{⊥}$($\varepsilon_{∥}$) measures the response for LC molecules for fields applied perpendicular (along) the nematic director field. Note that values of $\varepsilon_{⊥}$ and $\varepsilon_{∥}$ for temperatures $T>T_{c}$ are smaller (due to higher temperatures and finite sizes of paranematic clusters) but comparable to those in the nematic phase. With this in mind, it follows that on decreasing *T*, the relative contribution of $\varepsilon^{\left(c\right)}$ progressively dominates in ε and consequently ε(T)-slope gradually converts from $\frac{d\varepsilon }{dT}<0$ to $\frac{d\varepsilon }{dT}>0$.

On the contrary, in the nonlinear regime, the external field is strong enough to align $n^{\left(c\right)}$ of most of clusters along E. Consequently, in this regime the $\varepsilon^{\left(c\right)}$ response is dominated by $\varepsilon_{∥}$ contribution, where $\varepsilon_{∥}\gg \varepsilon_{⊥}.$ This yield monotonically increasing dielectric response on decreasing *T*. In the case of ODIC situation is much more complicated. Namely, both pretransitional clusters in translational and orientational order are expected.

## 4. Methods

In our study, we used the broadband dielectric spectrometer (BDS; Novocontrol), supported by the strong electric field facility enabling nonlinear dielectric spectroscopy studies and the Quattro temperature control unit [40]. Samples were placed in the flat-parallel measurement capacitor with plates made from Invar and gold-coated: diameter 2r=20 mm and the gap d=0.1 mm. Scans of dielectric properties were carried out in the frequency range 0.1 Hz<f<10 MHz under the weak measuring voltage $U_{weak}=1$ V, corresponding to the electric field $E_{weak}=14$ kV/m. The scan of dielectric properties under the strong electric field was carried out for $U_{strong}>1000$ V ($E_{strong}>5$ MV/m), limited to f<10 kHz [38,43].

The strong electric field related counterpart of dielectric constant is the nonlinear dielectric effect (NDE) [54,55,56]: $\varepsilon \left(E\right)=\varepsilon \left(E\to 0\right)+\Delta \varepsilon E^{2}+\ldots$, where ε(E→0)=ε represents the dielectric constant and for the nonlinear dielectric effect metric:

(16) $$NDE≔\frac{∆\varepsilon }{E^{2}}=\frac{\varepsilon \left(E\right)-\varepsilon }{E^{2}}$$

**Figure 6:**
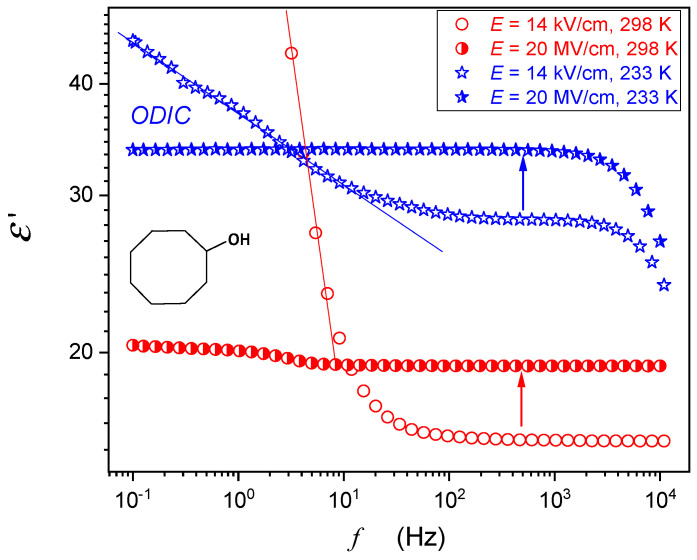
The real part of dielectric permittivity ($\varepsilon^{\prime }$) determined under the weak and the strong electric field in the (isotropic) liquid (red, circles) and the plastic crystal ODIC phase (blue, stars) in cyclooctanol. Horizontal parts in the plot yield dielectric constant ε related static domain. The schematic sketch of cyclooctanol’s structure is also shown.

NDE was calculated using Equation (16) from dielectric constant values in the middle of the static domain from $\varepsilon^{\prime }\left(f,E\right)$ spectra as shown in Figure 6. 

As the representative ODIC-type system we chose the cyclooctanol (C_8_H_16_O). Dielectric measurements were carried out in the extreme range of temperatures 120 K<T<345 K. Cyclooctanol is one of the most classical glass-forming materials, exhibiting the I-PC melting at $T_{m}\approx 278-292.5$ K, and subsequently the vitrification, for which the glass transition temperature $T_{g}$ is reported within the temperature window from 150 to 240 K [24,25,26,27,28,29]. The material with the highest declared purity was purchased from Sigma-Aldrich. It was additionally dried using 4 Å molecular sieves.

Tests focused on the real part of dielectric permittivity and dielectric constant have been hardly carried out for ODIC-forming materials so far. Examples of $\varepsilon^{\prime }\left(f\right)$ spectra under the weak and strong electric fields are shown in Figure 1. The horizontal parts determine the dielectric constant: $\varepsilon =\varepsilon^{\prime }\left(f\right)$. Under the weak electric field there is a notable impact of ionic dopants related to the notable increase for lower frequencies (f<100 Hz), reflecting the Maxwell–Wagner process [44]. This phenomenon disappears under a strong electric field. The slowing down on cooling towards the glass transition causes that for the ODIC phase the impact of the high frequency relaxation process becomes detectable at lower and lower frequencies. 

As an LC representative system, we used octyloxycyanobiphenyl (8OCB). It consists of rod-like molecules with a relatively large permanent dipole moment μ≈6.6 D parallel to the long molecular axis. It exhibits the following mesomorphism: isotropic liquid (I) (352.7 K) to nematic (N) (339.8 K) to Smectic A (SmA) (322.1 K) to crystal (Cr). It belongs to the group of classical LC compounds, and it is included to LC mixtures for displays applications [5,6].

## 5. Conclusions

The advanced way of dielectric constant determination from BDS spectra matched with the extreme range of temperatures studied (120 K < *T* < 350 K, for 220 selected temperatures) and supported nonlinear dielectric effect studies led to the qualitatively new insight into the physical properties of ODIC-forming cyclooctanol. Such unique experimental results led evidence a well-defined pretransitional effect both for the isotropic liquid-ODIC transition and within the ODIC phase. To confirm these results additional NDE measurements have been carried out using the dual field measurement principle, being the extreme resolution method associated with strong dielectric field pulses limited to only a few milliseconds. 

The analysis of the reciprocal of dielectric constant vs. temperature showed that ODIC-forming materials, both in the liquid and ODIC phase, can constitute the system were the Mossotti catastrophe [50,56] is the experimental fact, not only the interesting speculative paradox. Consequently, the question arises of designed studies on ODIC-forming systems can lead to a new type of ferroelectric material? 

Finally, this report indicates the possible significance of studies in symmetry-selected systems for approaching the cognitive breakthrough for the puzzling case of discontinuous phase transitions.

## Figures and Tables

**Table 1:** Values of parameters describing *NDE* vs. *T* experimental data in the liquid and ODIC phases of cyclooctanol (Figure 6) when portraying by Equation (8).

Transition→ Parameters ↓	ODIC→postODIC	ODIC→I	I→ODIC
$\varepsilon^{*}$	−10.930	5.467	−1.630
*A*	−0.556	0.138	−0.160
*B*	5.682	−1.205	1.963
$T^{*}$ (K)	220.0	293.9	276.0
φ	0.5	0.5	0.5
$T_{m}$ (K)	232.4	287.2	287.2
$\Delta T^{*}$ (K)	12.4	6.7	11.2

## Data Availability

The data presented in this study are available on a request from a corresponding author.

## Supplementary Materials

The following are available online, Minimal models comparing liquid crystalline and plastic crystal phase behavior and derivation.

## Sample Availability

Samples of the compounds are not available from the authors.

## Appendix A. Claussius-Mossoti Equation

Here, we recall the derivation of the classical Claussius-Mossoti equation which relates macroscopic dielectric response, represented by ε, with microscopic dipolar properties, represented by the dipolar polarizability α [42,52,53]. We consider a spatially homogeneous dielectric system, whose total electric dipole polarization P equals zero in the absence of an external electric field E. For the sake of simplicity, we represent the dielectric response with the scalar (i.e., orientationally independent) dielectric constant ε. In the presence of E, the macroscopic polarization appears as

(A1) $$P=\varepsilon_{0}\left(\varepsilon -1\right)E.$$

Furthermore, it holds that ~np, where p stands for an average electric dipole and *n* is the volume density of dipoles. For relatively weak external dielectric fields, one assumes a linear-type response

(A2) $$p\sim \alpha E_{loc},$$

where α is the polarizability constant, and

(A3) $$E_{loc}\sim E+\frac{P}{3\varepsilon_{0}}$$

is the local field acting on p. By combining Equation (A1), Equation (A2), and Equation (A3) one obtains the classical Claussius-Mossotti equation:

(A4) $$\frac{\varepsilon -1}{\varepsilon +2}=\frac{N\alpha }{3\varepsilon_{0}}\;,$$

which relates ε and α.

The Lorentz–Lorentz local field (Equation (A3)) leads to the relation:

(A5) $$\varepsilon -1=\frac{N\alpha /\varepsilon_{0}}{1-N\alpha /3\varepsilon_{0}}$$

The Lorentz–Lorentz local field and the Clausius–Miosotti equation are for non-polar dielectrics, for which the catastrophe emerging from Equation (A5) has to be absent. However, the catastrophe can appear for dipolar dielectrics where $\alpha =\alpha_{e}+\alpha_{a}+N\mu^{2}/3k_{B}T$, which yields the relation

(A6) $$\varepsilon -1=\frac{3T_{C}}{T-T_{C}}$$

with $T_{C}=\left(N\mu^{2}/9\varepsilon_{0}k_{B}\right)\left[1/\left(1-N\left(\alpha_{e}+\alpha_{a}\right)/3\varepsilon_{0}\right)\right]$.

The Mosotti Catastrophe suggests the possible Curie-Weiss pretransitional behavior for classical non-ferroelectric dielectrics. For instance, for water Equation (A6) leads to the para- to ferroelectric phase transition and solidification at $T_{C}\approx 1520K$, in clear disagreement with the nature. To overcome the paradox, more realistic local fields were developed [42,52,53].

However, the question arises what happens if a system of non-interacting or weakly interacting permanent dipole moment appears to be experimentally accessible? Is this the case of ODIC-forming materials?
