# Supplementary material for: Pretransitional Effects of the Isotropic Liquid–Plastic Crystal Transition
Source: Molecules. 2021 Jan 15;26(2):429. doi: 10.3390/molecules26020429 (PMC7830473; doi:10.3390/molecules26020429)
Supplement: Supplementary file 1 [file molecules-26-00429-s001.pdf]

Supplementary Materials

Pretransitional effects of the isotropic liquid-plastic crystal transition

A. Drozd-Rzoska, S. Starzonek, S. J. Rzoska, J. Łoś, Z. Kutnjak, S. Kralj

ODIC MINIMAL MODEL

Below we propose a minimal model for temperature-driven liquid-plastic crystal-crystal phase transition sequence, which we henceforth refer as the ODIC model. We introduce it based on the analogous behavior in ordinary thermotropic liquid crystals, while in the ODIC case we reverse the phase transition sequence in which an orientational and translational order appear**.** We first recall a minimal model describing the thermotropic isotropic (I) - nematic (N) - smectic A (SmA) liquid crystal phase sequence on decreasing temperature. Then we present our minimal ODIC model.

Liquid crystals

We consider bulk thermotropic LCs, which exhibit on lowering temperature liquid (isotropic), orientational nematic (N) order, and smectic A (SmA) order. For this purpose, we use a simple Landau-de Gennes-type [1-3] uniaxial mesoscopic description. The uniaxial orientational nematic order is described by the uniaxial tensor nematic order parameter

$$\boldsymbol{Q} = S(\boldsymbol{n}\otimes\boldsymbol{n} - \boldsymbol{I}/3), \qquad \text{(S1)}$$

consisting of the uniaxial order parameter S and the nematic director field $\boldsymbol{n}$. Here $S \in [-1/2,1]$ reveals the degree of nematic ordering. Note that states $S = \pm 1$ are physically different, and S=0 fingerprints the isotropic order. The unit vector field $\boldsymbol{n}$ exhibits the head-to-tail invariance, i.e., the states $\pm\boldsymbol{n}$ are physically equivalent. The smectic A translational order is determined by the complex smectic order parameter

$$\psi = \eta e^{i\phi}. \qquad \text{(S2)}$$

The amplitude $\eta \geq 0$ measures the degree of translational order. The position of smectic A layers is determined by the phase ϕ. In the bulk nematic equilibrium, $S(\boldsymbol{r})$ and $\boldsymbol{n}(\boldsymbol{r})$ are spatially homogeneous, where $\boldsymbol{n}$ is pointing along an arbitrary symmetry-breaking direction. In the bulk equilibrium SmA phase, in addition to the spatially homogeneous nematic order, also a regular stack of smectic layers appears. It is characterized by a spatially homogenous value of $\eta(\boldsymbol{r})$ and the phase $\phi(\boldsymbol{r}) = q_0\boldsymbol{n}.\boldsymbol{r}$ determines position of layers of thickness $d_0 = \frac{2\pi}{q_0}$. Note that $\boldsymbol{n}$ points along a smectic layer normal, i.e. $\boldsymbol{n} = \frac{\nabla\phi}{|\nabla\phi|}$.

At the I-N and the N-SmA phase transition, a continuous symmetry is broken in orientational and translational order, respectively. Consequently, both order parameters fields $(\boldsymbol{Q}(\boldsymbol{r}), \psi(\boldsymbol{r}))$ consist of two qualitatively different contributions: the *amplitude* fields $(S(\boldsymbol{r}), \eta(\boldsymbol{r}))$ and the *symmetry breaking* (also referred to as the *gauge*) fields $(\boldsymbol{n}(\boldsymbol{r}), \phi(\boldsymbol{r}))$. The *amplitudes* reveal strengths of the established ordering, while the symmetry-breaking fields reveal symmetry breaking choice of a relevant phase transition. Consequently, in a bulk equilibrium phase, a relevant *amplitude* field exhibits unique value, while a relevant *gauge field* exhibits infinite degeneracy.

In terms of the nematic and smectic order parameters, we express the free energy $f = f_n^{(c)} + f_n^{(e)} + f_n^{(f)} + f_s^{(c)} + f_s^{(e)} + f_c$ as a sum of nematic and smectic contributions. These terms are (in the lowest order expansion necessary to describe a sequence of I-N and N-SmA phase transitions) commonly expressed as follows [4]:

$$f_n^{(c)} = a_n(T - T_n^*)S^2 - b_nS^3 + c_nS^4, \tag{S3a}$$

$$f_n^{(e)} = L|\nabla\boldsymbol{Q}|^2, \tag{S3b}$$

$$f_n^{(f)} = -\varepsilon_0\Delta\varepsilon S(\boldsymbol{n}.\boldsymbol{E})^2/2, \tag{S3c}$$

$$f_s^{(c)} = a_s(T - T_s^*)|\psi|^2 + b_s|\psi|^4 + \cdots, \tag{S3d}$$

$$f_s^{(e)} = C_\perp |(\boldsymbol{n} \times \nabla)\psi|^2 + C_\parallel |(i\boldsymbol{n}q_0 - \nabla)\psi|^2. \tag{S3e}$$

$$f_c = -DS\eta^2. \tag{S3f}$$

The quantities $a_n, b_n, c_n, T_n^*, a_s, b_s, T_s^*, D$ are positive material constants enabling a 1st order I-N and N-SmA phase transition. The character of the latter transition depends on D value which, determines the coupling strength between S and η. For D=0 the I-N phase transition takes place at $T_{IN} = T_n^* + b_n^2/(4a_nc_n)$ and the N-SmA transition at $T_{NA} = T_s^* < T_{IN}$. In this case the phase transitions are determined by the nematic ($f_n^{(c)}$) and smectic ($f_s^{(c)}$) condensation term, respectively. The nematic elastic term $f_n^{(e)}$ enforces a spatially homogeneous ordering of $\boldsymbol{Q}(\boldsymbol{r})$, where L stands for a positive representative nematic elastic constant. The term $f_n^{(f)}$ describes the coupling of an external electric field $\boldsymbol{E}$ with $\underline{Q}(\boldsymbol{r})$. For a positive field anisotropy $\Delta\varepsilon$ this term enforces parallel alignment of $\boldsymbol{n}$ and $\boldsymbol{E}$. The smectic elastic term $f_s^{(e)}$ is weighted by the positive smectic bend ($C_\perp$) and compressibility elastic constant ($C_\parallel$). The former tends to align a smectic layer normal along $\boldsymbol{n}$. Furthermore, the compressibility term enforces the smectic layer periodicity q_0. The coupling term f_c could quantitatively and also qualitatively affect LC phase behavior.

Minimal ODIC model

We next consider the simplest possible mesoscopic modeling of plastic crystals. We refer to the corresponding minimal approach as the ODIC model. In the modeling, we originate from the minimal Landau-de Gennes model which is designed to describe the sequence of phases, where first the nematic orientational order, and afterward additional translational order appears on decreasing temperature from the isotropic (liquid) phase. In the ODIC model, we assume the following sequence of 1st order phase transitions on decreasing temperature: liquid (L), plastic (P), crystal (C) phase. Long-range translational order is established at the L-P transition. In the subsequent P-C transition an additional orientational order also appears, which

we describe by a vector order parameter. Therefore, the sequence in which ordering in orientational and translational ordering appears are in these cases reversed. We also propose an explanation why the P-C transition is often replaced by a glass-type transformation.

Following LC example, we describe a translational ordering with the complex order parameter

$$\psi = m_t e^{i\phi}. \tag{S4}$$

The amplitude m_t measures the degree of translational ordering and the phase ϕ determines the translational structural ordering. For example, a structure exhibiting a simple periodic undulation determined by the wave vector $\boldsymbol{q} = q\boldsymbol{e}_t$ (where $|\boldsymbol{e}_t| = 1$), is described by

$$\phi = \boldsymbol{q}.\boldsymbol{r} = q\boldsymbol{e}_t.\boldsymbol{r}\ . \tag{S5}$$

We describe the orientational ordering with the vector

$$\boldsymbol{m} = m_o\boldsymbol{e}_o\ . \tag{S6}$$

where the amplitude m_o measures the degree of orientational ordering, and the unit vector $\boldsymbol{e}_o$ points along a locally selected direction.

In terms of these fields we describe the phase sequence liquid-plastic-crystal phase on reducing the temperature. These phases are characterized by $\{m_o = 0, m_t = 0\}$, $\{m_o = 0, m_t > 0\}$, and $\{m_o > 0, m_t > 0\}$, respectively. Using a standard Landau-type phenomenological approach we write the free energy density f as an expansion in order parameters ψ and $\boldsymbol{m}$, where symmetry allowed terms are considered. We express it as a sum $f = f_t + f_o + f_c$ containing only translational (f_t) and orientational (f_o) degrees of freedom, and the term (f_c) describing the coupling between these degrees. We further decompose $f_d = f_d^{(c)} + f_d^{(e)} + f_d^{(f)}$ (where the subscript $_d$ stands either for $_t$ or $_o$) into the sum of the condensation ($f_d^{(c)}$), elastic ($f_d^{(e)}$), external field ($f_d^{(f)}$) contribution. These terms are in the

lowest order expansion necessary to describe sequence of 1st order phase transitions L-P and P-C expressed as follows:

$$f_t^{(c)} = a_t(T - T_t^*)|\psi|^2 - b_t|\psi|^4 + c_t|\psi|^6, \tag{S7a}$$

$$f_t^{(e)} = \kappa_t|(i\boldsymbol{q}_0 - \nabla)\psi|^2, \tag{S7b}$$

$$f_t^{(f)} = -\chi_t^{(1)}|\psi|^2|\boldsymbol{E}_{eff}|^2 - \chi_t^{(2)}|\nabla\psi.\boldsymbol{E}_{eff}|^2, \tag{S7c}$$

$$f_o^{(c)} = a_o(T - T_o^*)|\boldsymbol{m}|^2 - b_o|\boldsymbol{m}|^4 + c_o|\boldsymbol{m}|^6, \tag{S7d}$$

$$f_o^{(e)} = \kappa_o|\nabla\boldsymbol{m}|^2, \tag{S7e}$$

$$f_o^{(f)} = -\chi_o\boldsymbol{m}.\boldsymbol{E}_{eff}, \tag{S7f}$$

$$f_c = -d_1|\boldsymbol{m}|^2|\psi|^2 - d_2|\boldsymbol{m}.\nabla\psi|^2. \tag{S7g}$$

The quantities a_t, b_t, c_t, a_o, b_o, c_o are positive material constants enabling a 1st order L-P and P-C phase transitions. If the order parameters are decoupled and in absence of external fields (i.e., $f_o^{(f)} = f_t^{(f)} = f_c = 0$), then T_t^* and T_o^* determine the supercooling liquid and plastic phase temperature, respectively. We set $T_o^* < T_t^*$. The translational elastic term for a positive translational elastic module κ_t enforces $\phi = \boldsymbol{q}_0.\boldsymbol{r}$ and uniform value of m_t, where the wave vector $\boldsymbol{q}_0$ determines the translational symmetry in the plastic phase. Note the difference between the translational elastic terms describing plastic and SmA phase ordering (see Eq. (S3e) and Eq. (S7b)). In LCs the nematic orientational order introduces a symmetry breaking direction along which translational order is established in the SmA phase. On the contrary in the plastic phase, the orientational order is absent. The quantity

$$\boldsymbol{E}_{eff} = \boldsymbol{E} + \boldsymbol{E}_{rf} = E_{eff}\boldsymbol{e}_{eff} \tag{S8}$$

stands for an effective electric field, which we decompose into an ordering ($\boldsymbol{E} = E\boldsymbol{e}_E$) and random field-type ($\boldsymbol{E}_{rf} = E_{rf}\boldsymbol{e}_{rf}$) component, and $\{\boldsymbol{e}_{eff}, \boldsymbol{e}_E, \boldsymbol{e}_{rf}\}$ are unit vectors. Here $\boldsymbol{E}$

determines a field contribution due to an applied external voltage to a cell confining a sample. If the constants $\chi_t^{(1)}$ and $\chi_t^{(2)}$ are positive, $\vec{E}$ promotes the formation of translational ordering. Due to symmetry consideration these terms are in the lowest order proportional with E_{eff}^2. The term weighted with $\chi_t^{(1)}$ ($\chi_t^{(2)}$) enforces isotropic (anisotropic) translational ordering if $\boldsymbol{E} \neq 0$. In cases, where a crystal phase is replaced by a short-ranged glass-like phase, we assume a strong enough value of $\boldsymbol{E}_{rf}$. The latter contribution could arise due to spatially nonuniform orientation of molecular electric dipoles within a system. Note that in general both the amplitude $\boldsymbol{E}_{\mathrm{eff}}$ and $\boldsymbol{e}_{eff}$ might exhibit random variations.

A positive orientational elastic module κ_o favors homogeneous (i.e. m_o is spatially uniform) and uniform orientational ordering along a symmetry breaking direction. The vector character of the orientational order parameter allows linear coupling with external field. The constant χ_o determines the coupling strength with an effective electric field $\boldsymbol{E}_{eff}$. For a positive value, it tends to locally align $\boldsymbol{m}$ along the field direction.

The term f_c couples translational and orientational degrees of freedom. Due to symmetry requirements, it is in the lowest order expansion in order parameters proportional to $m_o^2 m_t^2$. We assume that both coupling constants d_1 and d_2 are positive, promoting mutual appearance of both degrees of ordering. The term proportional to d_1 (d_2) is isotropic (anisotropic).

We next consider a case when a constant external electric field E is applied and focus on the E-driven pretransitional response. We set that the coupling of E with the orientational degrees is stronger than with the translational order. Consequently, we neglect the free energy contribution given by Eq. (A7c). We also assume that $E \gg E_{rf}$. We set the spatially homogeneous order parameter. Furthermore, we assume that the dielectric response is dominated by orientational order, consequently we focus on $m_o(T)$ behavior.

For $E>0$ the most important free energy density f contributions for temperatures $T > T_o^*$ are

$$f \sim (a_o(T - T_o^*) - m_t^2(d_1 + d_2 q_0^2)\,)m_o^2 - \chi_o E m_o\ . \tag{S9}$$

Minimization of f with respect to m_o yields

$$m_o = \frac{\chi_o E}{2a_o\left(T - T_o^{(eff)}\right)}\ \ , \tag{S10}$$

where $T_o^{(eff)} = T_o^* + m_t^2(d_1 + d_2 q_0^2)/a_0$. For relatively weakly coupled order parameters it holds $T_o^{(eff)} = T_o^*$ for $T > T_t^{(c)}$, where $T_t^{(c)} = T_t^* + b_t^2/(4a_t c_t)$ determines the plastic-liquid phase transition temperature for E=0.

On the other hand for $T > T_o^{(c)}$, where $T_o^{(c)} = T_o^* + b_o^2/(4a_o c_o)$ determines the plastic crystal phase transition temperature for E=0, it roughly holds $T_o^{(eff)} \sim T_o^* + \frac{b_t(d_1 + d_2 q_0^2)}{2c_t a_o}$. Therefore, $T_o^{(eff)}$ has different values in the temperature regimes $T_t^{(c)} > T > T_o^{(c)}$ and $T > T_o^{(c)}$. Next we discuss conditions for which glass-type structures are expected. In the analysis above we neglected presence of random fields. These are in our treatment mimicked by $\boldsymbol{E}_{rf} = E_{rf}\boldsymbol{e}_{rf}$, see Eq. (S8). Their presence might have strong effects on the P-C phase transition. Namely, this transition corresponds to the continuous symmetry breaking in orientational ordering, which is directly coupled with $\boldsymbol{E}_{rf}$ (see Eq. (S7f)). According to the Imry-Ma theorem [5, 6], one of the pivotal theorems of statistical mechanics of disordered systems, even an infinitesimally weak random field-type disorder breaks long-range order due to the presence of Goldstone fluctuations in $\boldsymbol{m}$. These are inevitable present due to the continuous symmetry breaking. The resulting configurations are expected to exhibit short-range order and well-characterized by a single domain length. However, recent studies [6] suggest that finite field strength is needed to establish short-range order in systems of our interest.

REFERENCES

1. P. G. de Gennes, J. Prost, *The Physics of Liquid Crystals* (Oxford Univ. Press, Oxford, 1995).

2. S. Chandrasekhar, *Liquid Crystals* (Cambridge Univ. Press., Cambridge, 2010).
3. J. W, Goodby, P.J. Collins, T. Kato, C. Tschierske, H. Gleeson, P. Raynes, V. Vill, *Handbook of Liquid Crystals* (Wiley, NY, 2014).
4. P. G. de Gennes, *Simple Views on Condensed Matter* (World. Sci., Singapore, 1992).
5. Y. Imry, S. Ma, *Random-Field Instability of the Ordered State of Continuous Symmetry*. Phys. Rev. Lett. **35**, 1399 (1975).
6. A. Ranjkesh, M. Ambrožič, S. Kralj, T. J. Sluckin, *Computational Studies of History Dependence in Nematic Liquid Crystals in Random Environments*, Phys. Rev. E **89**, 022504 (2014).

TABLE OF SELECTED SYMBOLS

**Symbol**	**Definition**
T_m	melting temperature
T_g	glass transition temperature
T_{I-N}	isotropic-nematic phase transition temperature
T^*	ideal phase transition temperature
ΔT^*	phase transition discontinuity
ε^*	dielectric constant at T*
T_C	critical temperature
T^+	ideal phase transition temperature for ODICs
α, β, γ	universal critical exponents
